# mRNA therapy for myocardial infarction: A review of targets and delivery vehicles

**DOI:** 10.3389/fbioe.2022.1037051

**Published:** 2022-11-25

**Authors:** Xinming Wang, Douglas H. Wu, Samuel E. Senyo

**Affiliations:** ^1^ Department of Cardiovascular Surgery, Ruijin Hospital, Shanghai Jiaotong University School of Medicine, Shanghai, China; ^2^ Department of Biomedical Engineering, Case Western Reserve University, Cleveland, OH, United States

**Keywords:** mRNA therapy, myocardial infarction, delivery vehicle, clinical trials, lipid nanoparticle

## Abstract

Cardiovascular diseases are the leading cause of death in the world. This is partly due to the low regenerative capacity of adult hearts. mRNA therapy is a promising approach under development for cardiac diseases. In mRNA therapy, expression of the target protein is modulated by delivering synthetic mRNA. mRNA therapy benefits cardiac regeneration by increasing cardiomyocyte proliferation, reducing fibrosis, and promoting angiogenesis. Because mRNA is translated in the cytoplasm, the delivery efficiency of mRNA into the cytoplasm and nucleus significantly affects its therapeutic efficacy. To improve delivery efficiency, non-viral vehicles such as lipid nanoparticles have been developed. Non-viral vehicles can protect mRNA from enzymatic degradation and facilitate the cellular internalization of mRNA. In addition to non-viral vehicles, viral vectors have been designed to deliver mRNA templates into cardiac cells. This article reviews lipid nanoparticles, polymer nanoparticles, and viral vectors that have been utilized to deliver mRNA into the heart. Because of the growing interest in lipid nanoparticles, recent advances in lipid nanoparticles designed for cardiac mRNA delivery are discussed. Besides, potential targets of mRNA therapy for myocardial infarction are discussed. Gene therapies that have been investigated in patients with cardiac diseases are analyzed. Reviewing mRNA therapy from a clinically relevant perspective can reveal needs for future investigations.

## 1 Introduction

**FIGURE 1:**
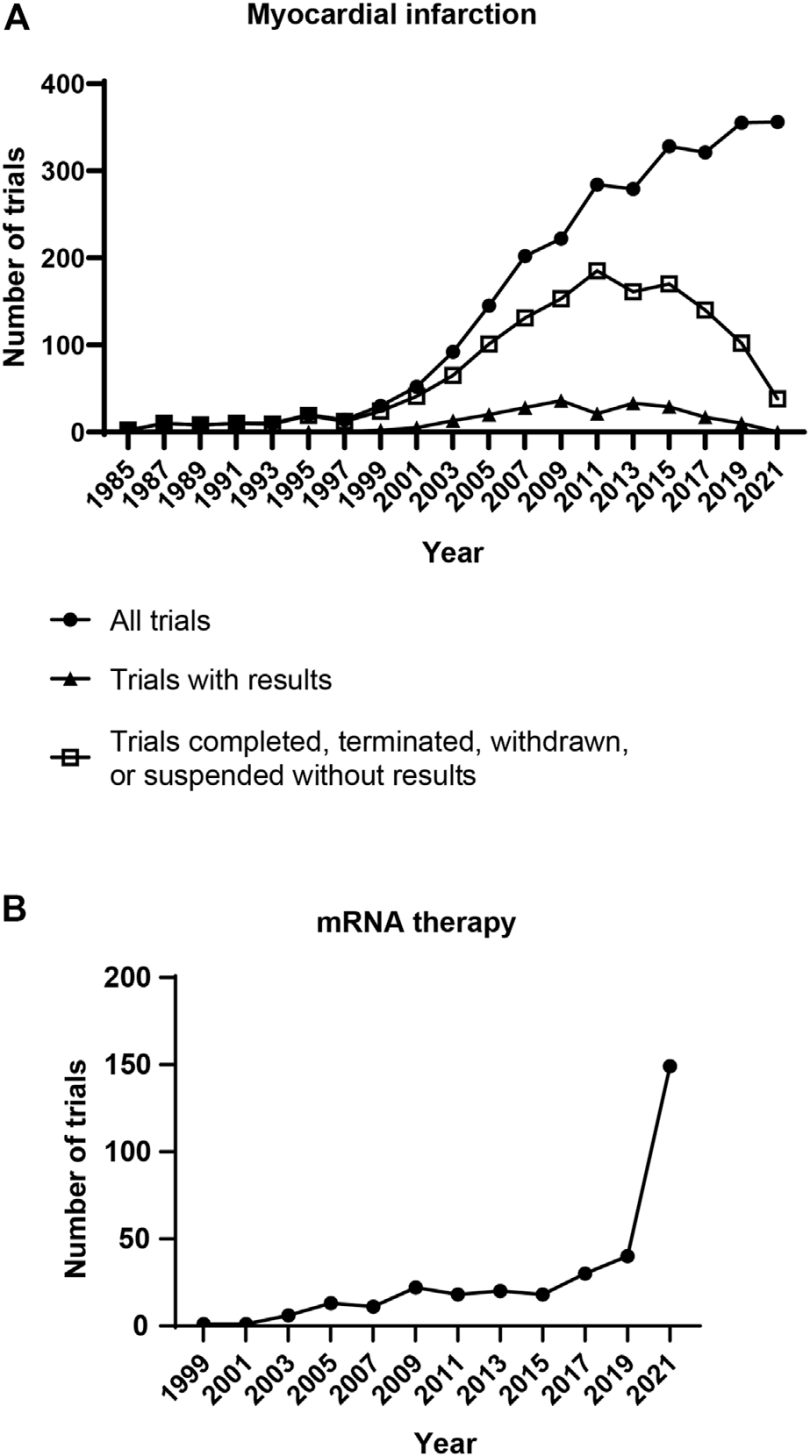
Clinical trials related to mRNA therapy and myocardial infarction. **(A)** Number of clinical trials for myocardial infarction. Each dot represents the clinical trials initiated in the following 2 years. Clinical trials have been rising for 36 years since 1985. However, only a small portion of trials disclosed the results. **(B)** Number of clinical trials for mRNA therapy. The clinical trials related to mRNA therapy have been rising for 22 years. Notably, a burst growth of clinical trials appeared in 2020, the year of the COVID-19 pandemic. [Data were collected from ClinicalTrials.gov (NIH, U.S. National Library of Medicine) on 22 April 2022. For panel A, trials were searched using the words “myocardial infarction” in the *Condition or disease* search field. All trials, trials with results, as well as completed, terminated, suspended, and withdrawn trials without results were downloaded for further analysis. For panel B, trials were searched using the words “mRNA therapy” in the *Intervention/treatment* search field, the study type was restricted to *Interventional Studies*. All trials were downloaded for further analysis. The distributions of start date were analyzed].

Cardiovascular diseases are a group of diseases related to heart muscles, blood vessels, and valves. The death caused by cardiovascular diseases worldwide in 2019 was 17.9 million, which accounts for approximately 30% of total death in the year (DofE and SAPD, 2019; World Heath Organization, 2021). Myocardial infarction and strokes result in over 80% of deaths from cardiovascular diseases. Percutaneous coronary intervention treatment has significantly lowered mortality after acute myocardial infarction. However, the cardiac function will be permanently impaired. Newborn mammals can regenerate the injured heart, but this regenerative capacity disappears in adults (Porrello et al., 2011; Ye et al., 2018). The declined regenerative capacity in aged hearts is partly due to decreased cardiomyocyte proliferation, lowered angiogenesis, and increased fibrosis (Rivard et al., 1999; Senyo et al., 2012; Notari et al., 2018). Cardiac regeneration is regulated by physical properties and biological factors (Bersell et al., 2009; Bassat et al., 2017; Wang et al., 2020; Wang et al., 2021a; Wang et al., 2021b). Delivering extracellular matrix biomolecules to stimulate cardiomyocyte division and heart angiogenesis promotes cardiac function in animal models of cardiac injury (Hao et al., 2007; Kühn et al., 2007; Wei et al., 2015; Rodness et al., 2016). Lowering microenvironment rigidity and fibrotic activity also benefit heart regeneration (Shao et al., 2006; Wang et al., 2010; Notari et al., 2018; Wang et al., 2021b). The growing understanding of cardiac regeneration has led to increased numbers of clinical trials for myocardial infarction in the past two decades (Figure 1A). Nevertheless, no therapy has revealed satisfying improvements in cardiac function and viability. Thus, developing effective treatments for heart regeneration remains a big challenge for myocardial infarction.

Messenger RNA (mRNA) therapy is a potential treatment strategy for myocardial infarction. Protein translation mediated by exogenous mRNA was first investigated *in vivo* in 1990 (Wolff et al., 1990). The study indicated that injecting synthetic mRNAs into mice skeletal muscle results in the expression of target proteins. Since then, synthetic mRNAs have been developed as therapeutics for various diseases (Figure 1B) (Koido et al., 2000; Connolly et al., 2018; Cao et al., 2019; Walsh et al., 2020). In 2021, more than a billion doses of mRNA-based COVID-19 vaccine were administrated worldwide (Barda et al., 2021; Patone et al., 2021; Kadali et al., 2022). Clinical data have indicated that mRNA therapy is relatively safe and effective for humans. Synthetic mRNA is produced by *in vitro* transcription (IVT) of DNA template (Pardi et al., 2013). In the first study of cardiac mRNA therapy, delivering vascular endothelial growth factor (VEGF) mRNA into myocardium improved VEGF production and cardiac function in post-myocardial infarction mice (Zangi et al., 2013). In a follow-up study, intramyocardial injection of mRNA encoding VEGF increased ejection fraction from ∼47% to ∼53% in a swine myocardial infarction model (Carlsson et al., 2018). mRNA can also de-differentiate human primary fibroblasts to induced pluripotent stem cells (iPSCs), and iPSCs can then differentiate into cardiomyocytes (Kogut et al., 2018; Ahmed et al., 2020). mRNA-based therapeutics are categorized as gene therapy in Europe (Iglesias-Lopez et al., 2019). As a relatively new treatment for cardiac diseases, more preclinical and clinical studies are needed to develop mRNA therapy for myocardial infarction.

A major challenge in mRNA therapy is the low mRNA delivery efficiency. This is partly due to the rapid RNA degradation and the low cellular uptake. RNAs are naturally unstable and susceptible to ribonucleases (RNases)-mediated degradation. For instance, systemically delivered RNAs only last for a few days due to rapid clearance from the blood by the liver and kidney (Bartlett et al., 2007; Huang Y. et al., 2011). RNAs delivered into solid tissue are deactivated in a short period due to Toll-like receptors-mediated innate immune response and RNases-mediated cleavage (Hemmi et al., 2000; Koczera et al., 2016). The cellular uptake determines how many mRNAs in the environment can reach the translation machinery. The uptake is mediated by scavenger receptor-mediated endocytosis (Lorenz et al., 2011). Some of the endocytosed mRNA molecules escape from the endosome to the cytoplasm where they are translated to proteins. However, the mRNA escape rate is very low (Lorenz et al., 2011). To improve mRNA delivery efficiency, vehicles such as lipid nanoparticles and polyplex have been developed. *In vitro* experiments have indicated that encapsulating mRNA into a vehicle increased the amount of intact mRNA by more than 10,000 times compared to naked mRNA after incubating in serum (Dirisala et al., 2019). In addition, the mRNA expression level was 15-fold higher in the mRNA-vehicle group than in the naked mRNA group. Because of their high protective efficacy, delivery vehicles have been widely employed in studies of mRNA therapy.

The therapeutic efficacy of mRNA is determined by mRNA degradation, cellular uptake, endosomal escape, and release kinetics from the delivery vehicle in the cytoplasm (Garneau et al., 2007). Because only a small portion of delivered mRNAs are eventually translated in the cytoplasm, increasing the intracellular delivery efficiency can improve the therapeutic efficacy of mRNA therapy. This article reviews the non-viral vehicles and viral vectors for intracellular polynucleotide delivery. Besides, the potential targets of mRNA therapy for myocardial infarction are introduced. In order to understand the challenges and needs in clinical studies, the clinical trials of gene therapy for cardiac diseases are discussed. This article aims to provide cues for future designs of mRNA therapy for myocardial infarction.

## 2 Targets for mRNA therapy

Several targets have been identified for heart repair. They are regulators of angiogenesis, cardiomyocyte division, and fibrosis. Potential targets for cardiac mRNA therapy are introduced in this section (Table 1). Comprehensive reviews of targets for cardiac gene therapy can be found in other articles (Zachary and Morgan, 2011; Talman and Ruskoaho, 2016; Payan et al., 2020).

**TABLE 1:** Potential targets for myocardial infarction mRNA therapy.

Reagent	Animal	Dose	Delivery method	Treatment result	Category	Reference
Human recombinant VEGF	Mice	2.4 ng total	PLGA nanoparticle	LVFS: Control: ∼15% Treatment: ∼20%	Angiogenesis	Oduk et al. (2018)
Rat recombinant VEGF	Rat	300 ng total	Alginate microsphere patch	LVFS: Control: ∼20% Treatment: ∼30%		Rodness et al. (2016)
Basic FGF	Mice	14 ng total	Intraperitoneal injection	LVFS: Control: ∼48% Treatment: ∼57%		Rao et al. (2020)
Basic FGF	Rat	5 µg total	Poly(NIPAAm-co-PAA-co-BA) hydorgel	LVFS: Control: ∼25% Treatment: ∼30%		Garbern et al. (2011)
Acidic FGF	Mice	500 ng total	Poly(ethylene argininylaspartate diglyceride)	LVEF: Control: ∼37% Treatment: ∼47%		Wang et al. (2017)
Recombinant human HGF	Mice	160 µg total	Intravenous injection	LVEDP: Control: ∼11 mmHg Treatment: ∼4 mmHg		Nakamura et al. (2000)
CTGF human monoclonal antibody	Mice	10 µg/g, 2 injections	Intraperitoneal injection	LVEF: Control: ∼16.5% Treatment: ∼27.1%	Fibrosis	Vainio et al. (2019)
Pharmaceutical inhibitor for TGF-β receptor (Activin receptor-like kinase 5)	Rat	50 µg/g/day	Gavage	LVEF: Control: ∼43.7% Treatment: ∼51.8		Tan et al. (2010)
Recombinant agrin	Mice	1 µg total	Intramyocardial injection	LVEF: Control: ∼41.9% Treatment: ∼55.0%	Cardiomyocyte proliferation	Bassat et al. (2017)
NOTCH ligand (Jagged-1 mimic)	Rat	N/A	Peptide hydrogel	LVEF: Control: ∼48% Treatment: ∼75%		Boopathy et al. (2015)
Neuregulin-1, EGF-like domain	Mice	2.5 µg total	Intraperitoneal injection	LVEF: Control: ∼22% Treatment: ∼32%		Bersell et al. (2009)

LVFS, left ventricular fractional shortening; LVEF, left ventricular ejection fraction; LVEDP, left ventricular end-diastolic pressure.

### 2.1 Angiogenesis

Angiogenesis describes the budding and growth of vessels from pre-existing vascular structures. In hearts with permanent ischemia, stimulating angiogenesis to recover the blood supply in infarct area benefits heart repair. VEGF, fibroblast growth factor (FGF), and hepatocyte growth factor (HGF) are well-investigated targets for angiogenic therapy (Table 1).

VEGF is an important regulator of angiogenesis and endothelial function. The U.S. Food and Drug Administration (FDA) has approved several drugs targeting VEGF-regulated pathways. The VEGF family has at least seven members, and VEGF-A is the dominant regulator of angiogenesis among them. VEGF receptors belong to the receptor tyrosine kinases (RTKs) family. RTKs activate various signaling pathways including extracellular signal-regulated kinase (ERK), phosphatidylinositol-3-kinase/protein kinase B/mammalian target of rapamycin (PI3K/Akt/mTOR), and phospholipase C gamma (PLCγ) (Zhou et al., 2021). Activating these signaling pathways stimulates endothelial cell proliferation and migration, which are essential processes in vessel formation. Several pre-clinical studies have demonstrated that VEGF treatment improves cardiac repair. For example, delivering recombinant VEGF proteins using nanoparticles or patches improved cardiac function in rodent myocardial infarction models (Rodness et al., 2016; Oduk et al., 2018).

FGF stimulates VEGF expression in endothelial cells and stromal cells. FGF receptors activate a variety of signaling pathways including PLCγ, mitogen-activated protein kinase (MAPK), and src-homology collagen (Shc). Many of the FGF-regulated signaling pathways are related to angiogenesis. For instance, the Shc protein is crucial for VEGF production (Saucier et al., 2004). Because of its robust angiogenic effect, FGF has been investigated in clinical trials for angina (Table 2). In pre-clinical trials, delivering FGF into the heart promoted cardiac repair in rodent myocardial infarction models (Rao et al., 2020; Khosravi et al., 2021).

**TABLE 2:** Clinical trials of gene therapy for cardiac diseases.

Disease	Vector	Target	Delivery method	Start date	NCT number
Heart failure	AAV1	SERCA2a	Intracoronary injection	2007/03	NCT00454818
		SERCA2a	Intracoronary injection	2012/07	NCT01643330
		SERCA2a	Intracoronary injection	2013/12	NCT01966887
		SERCA2a	Intracoronary injection	2014/07	NCT00534703
		SERCA2a	Intracoronary injection	2015/04	NCT02346422
		SERCA2a	Intracoronary injection	2021/10	NCT04703842
	Adenovirus 5	ADCY6	Intracoronary injection	2010/07	NCT00787059
		ADCY6	Intracoronary injection	2019/06	NCT03360448
	DNA plasmid	VEGF	Intramyocardial injection	2008/07	NCT00279539
		SDF-1	Intramyocardial injection	2010/02	NCT01082094
		SDF-1a, VEGF-165, S100A1	Intracoronary injection	2018/04	NCT03409627
Ischemic heart disease	Adenovirus	VEGF-121	Intramyocardial injection	2009/05	NCT01174095
		VEGF-D	Intramyocardial injection	2010/01	NCT01002430
	DNA plasmid	VEGF-A165	Intramyocardial injection	2003/03	NCT00135850
		HGF	Intramyocardial injection	2007/01	NCT01422772
		HGF	Intramyocardial injection	2018/01	NCT03404024
Angina pectoris	Adenovirus	VEGF-121	Intramyocardial injection	2005/04	NCT00215696
		FGF-4	Intracoronary injection	2007/05	NCT00438867
		FGF-4	Intracoronary injection	2012/03	NCT01550614
	DNA plasmid	VEGF-C	Intramyocardial injection	2004/08	NCT00090714
Myocardial infarction	DNA plasmid	HGF	Intramyocardial injection	2015/03	NCT01002495
	ASO	Lipoprotein a	Subcutaneous injection	2021/10	NCT04993664
Coronary artery disease	Adenovirus	VEGF	Intramyocardial injection	2020/01	NCT04125732
	DNA plasmid	VEGF-A165/bFGF	Intramyocardial injection	2004/12	NCT00620217
Atrial Fibrillation	ASO	CRP	Subcutaneous injection	2012/10	NCT01710852
	Adenovirus	KNCH2	Painting polymer hydrogel on epicardium	2022/09	NCT05223725
Crigler-Najjar Syndrome	AAV8	UGT1A1	Intravenous injection	2017/09	NCT03223194
		UGT1A1	Intravenous injection	2018/03	NCT03466463
Danon disease	AAV9	LAMP2	Intravenous injection	2019/04	NCT03882437
Fabry Disease, cardiac variant	AAV2	α-Galactosidase A	Intravenous injection	2021/08	NCT05039866
Cardiomyopathy associated with Friedreich’s ataxia	AAV	Frataxin	Intravenous injection	2022/05	NCT05302271
Refractory angina, coronary artery disease	Adenovirus	VEGF-D	Intramyocardial injection	2019/10	NCT03039751
Atherosclerosis	ASO	apolipoprotein B	Subcutaneous injection	2012/08	NCT01598948
Transthyretin Amyloid Cardiopathy	ASO	Transthyretin	Subcutaneous injection	2021/06	NCT04843020

The table summarizes the clinical trials registered on ClinicalTrials.gov as of 21 April 2022. SERCA2a, Sarco/endoplasmic reticulum Ca^2+^ adenosine triphosphatase-2a; ADCY6, Adenylyl cyclase 6; VEGF, Vascular endothelial growth factor; SDF-1, Stromal-cell derived factor 1; S100A1, S100 Calcium Binding Protein A1; HGF, Hepatocyte growth factor; FGF, Fibroblast growth factors; CRP, C-reactive protein; KNCH2, Potassium voltage-gated channel subfamily H member 2; UGT1A1, UDP glucuronosyltransferase family 1 member A1; LAMP2, Lysosome-associated membrane protein 2 isoform B; AAV, Adeno-associated virus vector; ASO, Antisense oligonucleotides.

HGF is a potent angiogenic factor. It is a ligand of c-Met protein which activates PI3K and MAPK proteins. MAPK and PI3K are important nodes in VEGF-induced angiogenesis. HGF stimulates angiogenesis by stimulating VEGF expression and increasing endothelial cell proliferation and motility (Xin et al., 2001). *In vivo* study has indicated that HGF treatment improves cardiac function in post-infarction hearts (Nakamura et al., 2000).

### 2.2 Fibrosis

Cardiac fibrosis is characterized by excessive fiber protein deposition in the extracellular space. Ischemic heart injury and pressure overload are major reasons for cardiac fibrosis (Fan et al., 2012; Kong et al., 2013). After myocardial infarction, the inflammatory response triggered by cardiomyocyte cytokines stimulates fibroblast to myofibroblast differentiation (Kong et al., 2013). Myofibroblasts are alpha-smooth muscle actin (α-SMA)-rich cells that produce extracellular matrix fiber proteins (Ma et al., 2014; van den Borne et al., 2010). Fibroblast differentiation is mediated by profibrotic cytokines such as transforming growth factor beta (TGF-β) and connective tissue growth factor (CTGF) (Table 1) (Gore-Hyer et al., 2002; Cucoranu et al., 2005; Bondi et al., 2010; Lipson et al., 2012). Controlling the pathways that regulate fibroblast differentiation and fiber protein deposition may reduce pathological fibrosis in the heart.

TGF-β has been accepted as the dominant regulator of fibrosis in various organs. TGF-β triggers the fibrotic process *via* TGF-β receptor 1 (TGFR1). Activated TGFR1 phosphorylates Smad proteins. The Smad complex then translocates to the nucleus and initiates the transcription of specific genes including collagen, fibronectin, and α-SMA (Meng et al., 2016). Due to the pleiotropic role of TGF- β in many biological processes, blocking TGF-β activity can cause severe adverse effects (Teixeira et al., 2020). Targeting downstream components in the TGF-β signaling pathway helps to attenuate the side effects. For example, neutralizing TGF-β using pan-antibody led to local inflammation and squamous cell carcinomas, while inhibiting TGF-β type I receptor kinase (ALK5) did not induce those side effects (Ling and Lee, 2011). Several inhibitors for TGF-β signaling pathway have been investigated in animal models of cardiac injury. These studies have demonstrated that lowering TGF-β activity reduces fibrosis and protects cardiac function in post-myocardial infarction hearts (Frantz et al., 2008; Nguyen et al., 2010; Tan et al., 2010).

CTGF is a 38 kDa extracellular matrix protein that exists in many tissues. Similar to TGF-β, CTGF is one of the primary regulators of fibrosis. A positive feedback loop has been observed between CTGF and TGF-β: CTGF stimulates the expression of TGF-β, and TGF-β in turn further increases CTGF secretion (Yang et al., 2010). The exact mechanism of CTGF-regulated fibrosis has not been fully understood. Structural analysis of CTGF suggests that it interacts with insulin-like growth factors, TGF-β, and integrin (Chen et al., 2020). Because changing CTGF activity reduces fibrosis without causing severe adverse effects, this protein has been investigated as a target of anti-fibrotic therapy. *In vivo* study has indicated that neutralizing CTGF by monoclonal antibody lowered fibrosis and enhanced cardiac function in mice post-myocardial infarction (Vainio et al., 2019). On the other hand, cardiac-restricted overexpression of CTGF resulted in increased left ventricular function in mice (Gravning et al., 2012). The results imply that CTGF has biphasic effects on cardiac repair. Further optimization of CTGF inhibition is required to achieve the best therapeutic outcomes.

### 2.3 Cardiomyocyte proliferation

Increasing cardiomyocyte proliferation remains a big challenge for regenerating adult mammalian hearts. More than a billion cardiomyocytes are affected during an acute ischemic event. Cardiomyocyte necrosis can be significantly lowered by reperfusion within 3–4 h of ischemia. However, the follow-up apoptosis leads to approximately 37% of cardiomyocytes dying in a year (Mani, 2008). Cardiomyocytes in adult hearts proliferate at an extremely low rate which is far from enough to replace the dead cells (Porrello et al., 2011; Senyo et al., 2012). The signaling pathways controlling cardiomyocyte proliferation have not been fully understood. So far, studies have suggested that NOTCH, hippo, and neuregulin-ErbB signaling pathways regulate cardiomyocyte proliferation (Table 1) (Zhao et al., 2020).

Yes-associated protein 1 (YAP) is an important node in the Hippo pathway. Down-regulating the hippo pathway increases the activity of YAP protein. The activated YAP then translocates to the nucleus to initiate the expression of various genes. In addition to biological cues such as the hippo pathway, YAP activity is also sensitive to mechanical stimuli (Chakraborty et al., 2017). A possible mechanism of the mechano-modulation is that microenvironment stiffness affects the organization of F-actins which lower YAP activity through their capping/severing proteins (Aragona et al., 2013). *In vivo* and *in vitro* studies have demonstrated that YAP is a key regulator of cardiomyocyte proliferation. For example, enhancing YAP activity *via* recombinant agrin protein, a hippo pathway inhibitor, stimulated cardiomyocyte proliferation and promoted cardiac function in post-myocardial infarction mice (Bassat et al., 2017).

Neuregulin-1 (NRG1) is a member of the epidermal growth factor family. NRG1 regulates cardiomyocyte proliferation through its tyrosine kinase receptors, ErbB2 and ErbB4 (Zhao et al., 1998). The activated ErbB dimer stimulates cell proliferation *via* Src, Shc, and PI3K signaling pathways (Wadugu and Kühn, 2012). The exact function of NRG1-ErbB in cardiomyocyte mitosis has not been understood. However, overexpressing ErbB increases cardiomyocyte division in postnatal mice (Bersell et al., 2009). In preclinical studies, delivering neuregulin-1 into myocardium preserved cardiac function in mice model of myocardial infarction (Bersell et al., 2009).

The NOTCH family contains 4 transmembrane receptors. After binding to their ligands, NOTCH receptors release the functional domains into the cytoplasm. The domains then translocate to the nucleus and regulate the transcription of target genes, including Wnt and Hey (Ferrari and Rizzo, 2014). Wnt and Hey proteins are regulators of cardiomyocyte differentiation. The NOTCH signaling pathway has been confirmed as a key regulator of embryonic cardiovascular development. In adult hearts, activating the NOTCH signaling pathway using extracellular matrix ligands preserved cardiac output in rats model of myocardial infarction (Gude et al., 2008). However, the exact function of NOTCH in cardiomyocyte proliferation remains unknown.

## 3 mRNA delivery vehicles

**FIGURE 2:**
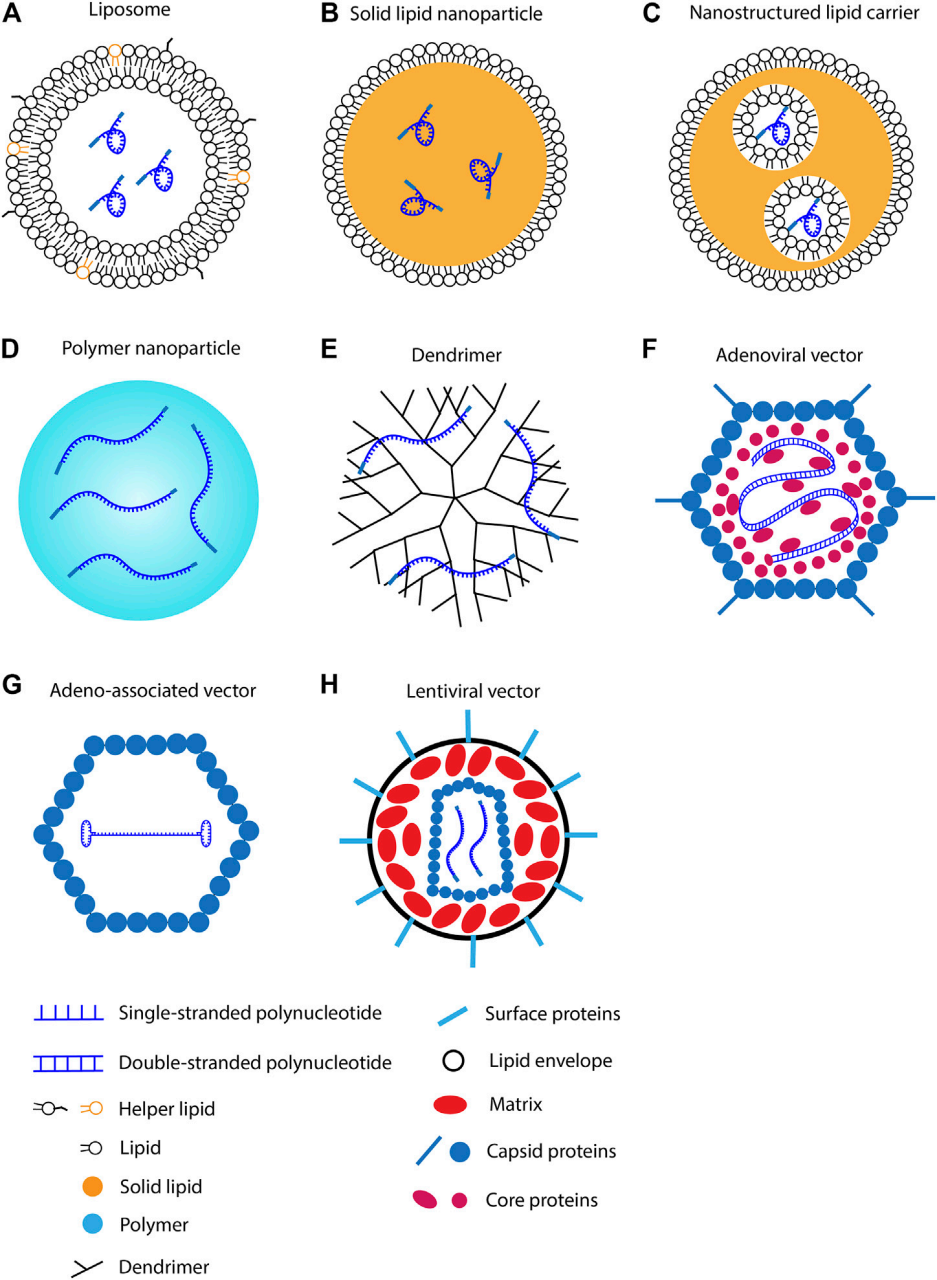
Methods for polynucleotide intracellular delivery. **(A)** RNA in lipid nanoparticle. **(B)** RNA in solid lipid nanoparticle. **(C)** RNA in nanostructured lipid carrier. **(D)** RNA in polymer nanoparticle. **(E)** RNA in dendrimer. **(F)** Double-stranded DNA in adenoviral vector. **(G)** Single-stranded DNA in adeno-associated viral vector. **(H)** RNA in lentiviral vector.

mRNA must enter the cytoplasm before directing protein synthesis. Various vehicles have been developed to improve delivery efficiency. The vehicles can be categorized into viral and non-viral vehicles (Figure 2). These vehicles are below 500 nm in diameter and thus are objects of endocytosis. Using these nano-scale vehicles has several advantages. First, the vehicles can be administrated *via* parenteral injection instead of surgical implantation. Second, active targeting of specific cells can be achieved by modulating the components of a vehicle. Third, vehicles could extravasate from the blood to the surrounding tissue. This process is mediated by transcytosis, enhanced permeability and retention (EPR) effect, and vascular leakiness (Li and Kataoka, 2021). Because of these advantages, nano-scale vehicles have become the dominant carrier of mRNAs for cardiac cells.

### 3.1 Lipid nanoparticles

Lipid nanoparticles are formed by lipid molecule self-assembly. mRNA can be encapsulated in lipid nanoparticles *via* electrostatic interactions. According to source materials, lipid nanoparticles can be categorized into several types. The most popular lipid nanoparticles for mRNA delivery are composed of cationic or ionizable lipids. Helper lipids are usually added to promote the stability of lipid nanoparticles. In addition, solid lipid nanoparticles have been developed for mRNA delivery but are relatively rare compared to liquid lipids nanoparticles.

#### 3.1.1 Cationic lipid nanoparticles

**FIGURE 3:**
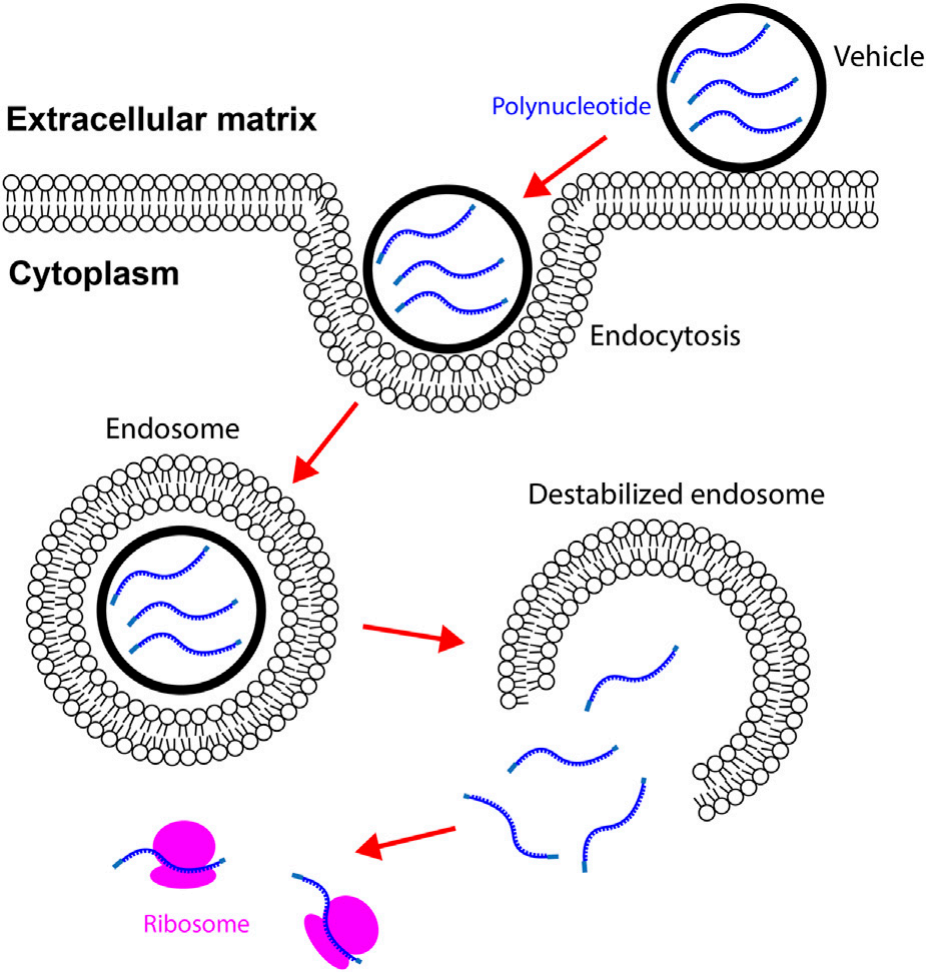
Vehicles carrying mRNAs enter the cytoplasm *via* endocytosis. Vehicles adhere to the cell membrane by binding to specific ligands or *via* electrostatic forces. Internalization of vehicles is mediated by endocytosis in cardiac cells. Vehicles destabilize endosomes by disrupting the integrity of endosome membrane. mRNAs in vehicles are then released to the cytoplasm. mRNAs are translated to proteins after binding to ribosomes.

Cationic lipid consists of a positively charged head group, a linker group, and hydrophobic tails. The negatively charged mRNA attracts cationic lipids to accumulate around its surface. Because mRNA utilizes the repulsive force to maintain the linear conformation, neutralizing the surface charge leads to mRNA folding (Draper, 2004). The collapsed mRNA molecule is then encapsulated by lipid bilayer to form a spheric nanoparticle (Figure 2A) (Gershon et al., 1993). After delivering into the body, lipid nanoparticles first adhere to the cell membrane and then be internalized *via* endocytosis (Vranic et al., 2013). Cationic lipid nanoparticles can rapidly fuse with the endosomal membrane and release mRNAs into the cytoplasm (Figure 3) (Zhou and Huang, 1994; Labat-Moleur et al., 1996). Cationic lipid nanoparticles have exhibited high RNA delivery efficiency *in vivo* and *in vitro* applications. Nevertheless, they can activate pro-apoptotic and pro-inflammatory signaling pathways (Cui et al., 2018).

**FIGURE 4:**
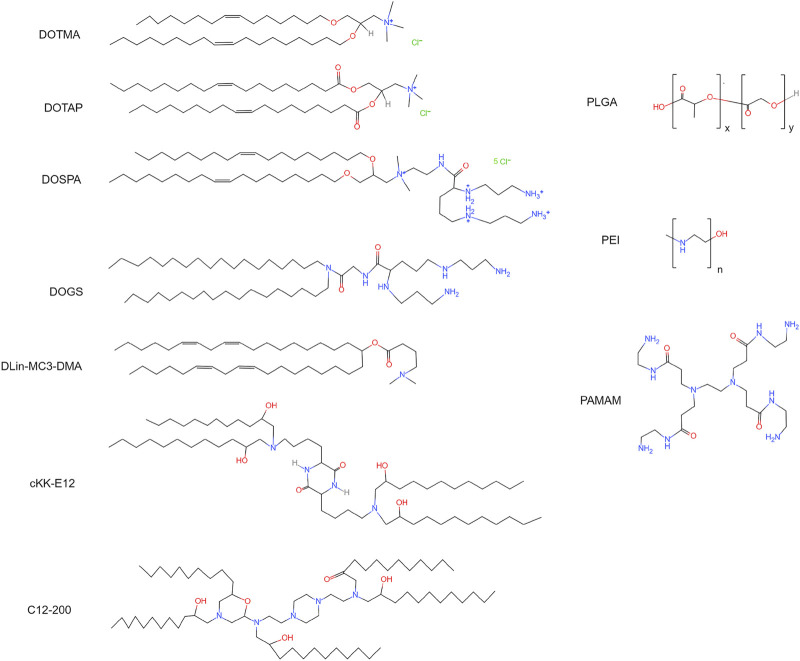
Chemical structures of cationic lipids, ionizable lipids, and polymers. DOTMA: 1,2-di-*O*-octadecenyl-3-trimethylammonium-propane. DOTAP: 1,2-dioleoyl-3-trimethylammonium-propane. DOSPA: 2,3-dioleyloxy-*N*-[2-(sperminecarboxamido)ethyl]-*N*,*N*-dimethyl-1-propanaminium trifluoroacetate. DOGS: 2,5-bis(3-aminopropylamino)-*N*-[2-[di(heptadecyl)amino]-2-oxoethyl] pentanamide. DLin-MC3-DMA: heptatriaconta-6,9,28,31-tetraen-19-yl-4-(dimethylamino) butanoate. cKK-E12: 3,6-bis(4-(bis(2-hydroxydodecyl)amino)butyl)piperazine-2,5-dione. C12-200: 1,1′-[[2-[4-[2-[[2-[bis(2-hydroxydodecyl)amino]ethyl](2-hydroxydodecyl)amino]ethyl]-1-piperazinyl]ethyl]imino]bis-2-dodecanol. PLGA, poly(lactic-co-glycolic acid); PEI, poly-ethylenimine; PAMAM, poly(amidoamine).

Polynucleotide delivery *via* cationic lipid nanoparticles has been investigated in animal models of cancer, immune disorder, infectious disease, and organ injury. Delivering cationic DOTMA (Figure 4) nanoparticles carrying antigen-encoding mRNAs into mice lung metastasis models resulted in clearance of metastases (Kranz et al., 2016). In a later study, the same DOTMA nanoparticle was used to deliver mRNAs encoding autoantigens into T cells. The treatment ameliorated autoimmune encephalomyelitis in a mice sclerosis model (Krienke et al., 1979). For regenerative medicine, studies of cardiac repair using cationic lipid nanoparticles carrying RNAs have shown promising results. Delivering VEGF mRNA *via* lipofectamine improved angiogenesis and cardiac repair *in vivo* (Zangi et al., 2013). Lipofectamine is a commercialized cationic lipid nanoparticle. In addition, intravenous injection of microRNA-19 encapsulated in lipofectamine preserved cardiac function in post-myocardial infarction mice (Gao et al., 2019). *In vitro* studies, lipofectamine effectively transfected iPSCs and iPSCs-derived cardiomyocytes (Warren et al., 2010; Tan et al., 2019). The transfected iPSCs can be employed for cardiogenic therapy. Together, cationic lipid nanoparticles are capable of mRNA delivery into mammalian cells, though the cell-specificity requires optimization.

#### 3.1.2 Ionizable lipid nanoparticles

Ionizable lipid nanoparticles are developed to overcome the limitations of cationic lipids (Meng et al., 2000; Hajj and Whitehead, 2017). Ionizable lipids usually contain amino head groups and their pK_a_ is below seven. These lipids are positively charged at low pH but neutrally charged at physiological pH. Thus, high RNA loading efficiency can be achieved when preparing ionizable lipid nanoparticles in an acidic environment. For example, lowering the pH from 6.5 to 3.5 increased the polynucleotide loading efficiency from ∼1% to ∼90% (Semple et al., 2001). The neutrally charged ionizable nanoparticles in the body have a low affinity to plasma proteins. As a result, these nanoparticles exhibit high affinity to the cellular membrane and increased half-life in the blood (Semple et al., 2001; Whitehead et al., 2014). Ionizable lipid nanoparticles are internalized by cells through endocytosis. Because the internal pH of endosomes is approximately 6.2, ionizable lipids are positively charged in endosomes. The positively charged lipid nanoparticles can easily interact with the negatively charged endosomal membrane. As a result of the interaction, the endosomal membrane is destabilized, and RNAs are released into the cytoplasm (Hafez et al., 2001). Due to the high RNA delivery efficiency, several ionizable lipids have been developed for RNA delivery, for instance, DOGS, DLin-MC3-DMA, and cKK-E12 (Figure 4).

Ionizable lipid nanoparticles have been widely utilized in preclinical studies to deliver polynucleotides. Injecting anti-transthyretin siRNA encapsulated by DLin-MC3-DMA reduced the transthyretin level by 60% in transthyretin amyloidosis patients (Adams et al., 2018). FDA approved this therapy in 2019. Other diseases, such as cancer and viral infection, can also be treated using ionizable lipid nanoparticles carrying RNAs. For example, delivering mRNAs encoding tumor-associated antigens encapsulated by cKK-E12 decreased tumor size and increased viability in mice cancer models (Oberli et al., 2017; Rybakova et al., 2019). Efficient mRNA delivery into cardiac cells using ionizable lipid nanoparticles has also been reported. C14-113 lipid nanoparticles have successfully delivered mRNAs encoding enhanced green fluorescent protein (EGFP) into cardiac cells in rats without causing observable side effects (Turnbull et al., 2016). C14-113 is a derivative of C12-200, an ionizable lipid (Figure 4) (Love et al., 2010). EGFP signal was observed for 6 h after injection. This study also demonstrated that intramyocardial injection results in heart-specific expression, while intravenous injection leads to the expression in various organs. Recent studies have indicated that ionizable lipid nanoparticle has a high mRNA delivery efficiency and relatively low cytotoxicity.

#### 3.1.3 Solid lipid nanoparticles (SLN) and nanostructured lipid carriers (NLC)

SLNs (Figure 2B) and NLCs (Figure 2C) are 40–1,000 nm spherical particles that are mainly composed of solid lipids (Naseri et al., 2015). The major difference between SLN and NLC is the inner structure. SLNs have a solid core; in contrast, NLCs have liquid phases in the core (Haider et al., 2020; Paliwal et al., 2020). SLNs were developed in the 1990s as a platform for sustained drug delivery (Muller et al., 1995). They have higher stability than liposomes, and thus are more suitable for long-term transportation and storage. Potential issues associated with SLNs are crystallization and low drug encapsulation efficiency (Mehnert and Mäder, 2001; Das and Chaudhury, 2011).

To overcome the limitations of SLNs, NLCs were developed as the advanced version of SLNs. NLCs are produced by blending solid lipids and liquid lipids at approximately 60°C (Pardeike et al., 2009). The resulting nanoparticles are solid at physiological temperatures but contain liquid lipids in the core. Compared to SLNs, NLCs exhibit increased loading capacity and lowered drug expulsion during storage (Mehnert and Mäder, 2001). Both SLNs and NLCs have relatively low cell toxicity. *In vitro* experiments have demonstrated that cells can tolerate 1 mg/ml solid lipids in the media (Doktorovová et al., 2016). However, more *in vivo* data are required to evaluate the adverse effects of solid nanoparticles.

SLNs and NLCs are capable of mRNA delivery. Intravenous injection of NLCs carrying EGFP genes resulted in the expression of EGFP for at least 74 h in mice (Han et al., 2016). Injecting NLCs carrying siRNAs lowered tumor growth and increased animal viability in mice cancer models (Chen et al., 2010; Costa et al., 2015; Yang et al., 2016). In addition, NLCs have successfully delivered mRNA-based vaccines into mice (Erasmus et al., 2018). SLNs and NLCs have been used to deliver chemical drugs into the heart. These nanoparticles delivered by intramyocardial injection protected the heart from ischemic injury in rodents (Tan et al., 2017; Alam et al., 2022). Although solid lipids have not been applied to mRNA delivery into cardiac cells, available studies suggest that SLNs and NLCs are potent for cardiac mRNA therapy.

#### 3.1.4 Helper lipids

Helper lipids can stabilize lipid nanoparticles and enhance delivery efficiency (Cheng and Lee, 2016). The common helper lipids are phospholipids, sterols, fatty acids, and PEG-lipids. Phospholipids are components of the natural cell membrane. Adding phospholipids to nanoparticles attenuates inflammatory response and facilitates endosome escape (Zuhorn et al., 2005). Sterols, such as cholesterol, are natural lipids in the animal cell membrane. Adding cholesterols into lipid nanoparticles increases stability in the blood and mRNA delivery efficiency (Zhigaltsev et al., 2002; Pozzi et al., 2012). Anionic fatty acid lipids are positively charged in endosomes (Yu et al., 2012; Wang et al., 2013). Increasing the positive charge on the nanoparticle surface facilitates mRNA release from the endosome. Polyethylene glycol (PEG)-lipids are important helper lipids. The lipid is composed of a hydrophilic PEG molecule and a hydrophobic alkyl chain. The PEG molecule prevents non-specific binding of plasma proteins to the surface of lipid nanoparticles (Yamamoto et al., 2016). Some of the plasma proteins, for example, opsonin, facilitate the capture and phagocytosis of lipid nanoparticles by macrophage. Thus, reducing the plasma protein-lipid nanoparticle affinity can increase the half-life of lipid nanoparticles in the blood. In an *in vivo* study, adding PEG-lipid increased the half-life of systemically delivered lipid nanoparticles from a few minutes to 5 h (Suzuki et al., 2020). Another important application of PEG-lipid is ligand conjugation. Conjugating ligands to PEG-lipid can significantly improve cell specificity. For example, covalently connecting folate to PEG-lipid increased the delivery efficiency by approximately 2.5 times in carcinoma cells (Krzysztoń et al., 2017).

#### 3.1.5 Advances in liposome-based cardiac RNA delivery techniques

The first generation of lipid nanoparticles was developed in 1965 (Bangham et al., 1965). Thirteen years later, lipid nanoparticles were employed as a vehicle for polynucleotide delivery into cells (Dimitriadis, 1978; Ostro et al., 1978). These studies indicated that incubating mammalian cells with lipid nanoparticles carrying mRNAs results in robust protein expression. In early 2020, the urgent need for COVID-19 vaccines led to rapid growth in clinical studies related to mRNA therapy (Figure 1). Eventually, in late 2020, mRNA vaccines for COVID-19 were commercialized. The vaccine is composed of a lipid nanoparticle vehicle and mRNAs encoding the SARS-CoV-2 full-length spike protein (Barda et al., 2021). Although encouraging progress has been achieved in mRNA-based therapies, the development of synthetic mRNA and mRNA delivery vehicles for cardiac diseases remains relatively slow. This sub-section focuses on lipid nanoparticles that have been employed for RNA delivery into the heart or cardiac cells in the past decade.

Delivering microRNA (miRNA) and small interfering RNA (siRNA) into the heart has been an important application of lipid nanoparticles. For example, intravenous injection of miR-145 encapsulated within lipid nanoparticles increased miR-145 levels in the plasma for approximately 30 min (Higashi et al., 2015). On day 2 after injection, the miR-145 level in the infarct area was 50% higher compared to the myocardial infarction-only group (Higashi et al., 2015). To improve the heart-specificity of lipid nanoparticles, ultrasound-guided lipid microbubbles were developed. The lipid microbubbles were filled with inert gas. The loaded drug, anti-miR-23a, adhered to the lipid bilayer *via* electrostatic force (Kopechek et al., 2019). After injection, ultrasound was applied to the heart to destruct lipid microbubbles (Fan et al., 2014). The dynamic response of microbubbles drove the formation of pores on the cell membrane, which enhanced cell membrane permeability to anti-miR-23a. Lipid microbubbles carrying anti-miR-23a significantly reduced the level of miR-23a in the heart compared to the negative control. In contrast, delivering naked anti-miR-23a failed to alter the level of miR-23a in the heart (Kopechek et al., 2019). Although miRNA has higher stability and lower molecular weight than mRNA, the lipid nanoparticles developed for miRNA can be utilized for mRNA delivery.

Cationic lipid-based nanoparticles have been a popular vehicle for polynucleotide delivery. In one of the earliest studies of mRNA therapy for cardiac diseases, VEGF mRNA was delivered into cardiac cells *via* Lipofectamine RNAiMAX (Zangi et al., 2013). In this study, mRNAs encapsulated by lipofectamine were injected directly into myocardium. This treatment significantly increased the expression of VEGF in the heart for 72 h. More than 80% of adult mouse cardiomyocytes, endothelial cells, and smooth muscle cells were transfected. Approximately 20% of cells died after transfection in *in vitro* experiments (Zangi et al., 2013). Interestingly, in a recent *in vivo* study, VEGF mRNA encapsulated in lipofectamine was injected into rodent hearts (Carlsson et al., 2018). In contrast, naked VEGF mRNA was delivered into the swine myocardium. Significantly improved ejection fraction was observed in mice on day 7, and in pigs on month 2. The reasons underlying this difference are unknown. Numerous cationic lipid nanoparticles have been employed for polynucleotide delivery into the heart in pre-clinical studies. However, none of them have been used in clinical studies due to hepatotoxicity, hypersensitivity reaction, and low specificity to organs other than the liver (Rudin et al., 2004). Thus, the field is still looking for lipid nanoparticles for mRNA delivery into human hearts.

Investigators have started to improve heart-specificity by modulating the components of lipid nanoparticles. A study conducted in 2019 has demonstrated interesting changes in lipid nanoparticle organ-specificity (Guimaraes et al., 2019). For lipid nanoparticles consisting of ionizable lipid, helper lipid, cholesterol, and PEGylated lipid (molar ratio 30: 16: 51: 3), the organ-specificity can be ranked as follow: brain ≥ lung ≥ heart > liver. Lowering the ratio of cholesterol (molar ratio 40: 16: 42.5: 1.5) changed the organ-specificity to liver ≥ heart > lung ≥ brain. However, none of the tested 16 formulas exhibited a significantly higher specificity to the heart than other organs (Guimaraes et al., 2019). A study conducted in 2022 used similar components for mRNA delivery into the heart. The molar ratio of ionizable lipid, helper lipid, cholesterol, and PEGylated lipid is 50: 10: 38.5: 1.5 (Evers et al., 2022). In this study, the mRNA delivery efficiency in post-ischemia hearts was approximately 4-times higher than that of sham hearts. A majority of the transfected cardiac cells were located in the infarct area, and most of the cells were fibroblast. Notably, approximately 200-times more mRNAs were accumulated in livers compared to ischemic hearts, though H&E staining revealed no observable liver damage. Thus, further increasing the heart-specificity of lipid nanoparticles may require methods other than adjusting the components.

Surface modification of lipid nanoparticles has been demonstrated to improve the therapeutic efficacy of mRNA therapy for cardiac injury. In a study published in 2022, CD5 antibodies were conjugated to the surface of lipid nanoparticles *via* SATA-maleimide chemistry (Rurik et al., 2022). CD5 is expressed mainly by T cells. The modification increased the mRNA delivery efficiency to T cells by approximately 20-times. The group then designed an mRNA encoding a marker of activated fibroblasts. The modified lipid nanoparticles carrying this mRNA activated fibroblast-specific chimeric antigen receptor (CAR) T cells. In a mouse hypertensive cardiac injury model, the targeted mRNA therapy significantly reduced the fibrotic area in the heart and preserved cardiac function (Rurik et al., 2022). Notably, almost all the transfected cells were in the liver and spleen after systemic delivery. This is probably due to hepatic clearance of lipid nanoparticles (Akinc et al., 2010). The study suggests that targeted mRNA delivery using surface-modified lipid nanoparticles can improve heart repair. Numerous surface modification methods have been developed to improve the cell-specificity of lipid nanoparticles, for example, increasing the surface charge and conjugating ligands. Detailed reviews of lipid nanoparticle surface modification can be found in other articles (Kularatne et al., 2022; Xu et al., 2022). These methods may provide cues for designing heart-specific lipid nanoparticles.

### 3.2 Polymer vehicles

Polymer biomaterials have been widely utilized in tissue engineering. Polymer substrates have controllable drug release rates and drug loading capacities. Although polymer mRNA delivery vehicles are not as common as lipid nanoparticles, they may provide a solution for sustained mRNA delivery.

#### 3.2.1 Liner polymer nanoparticles

Polymer nanoparticles are natural or synthetic compounds that form complex structures for drug delivery (Figure 2D). The polymers employed for polynucleotide delivery are usually positively charged so they can encapsulate RNA through electrostatic forces, for example, poly(lactic-co-glycolic acid) (PLGA) and poly-ethylenimine (PEI) (Figure 4). Polymer nanoparticles are internalized by cells *via* endocytosis, micropinocytosis, or phagocytosis (van der Aa et al., 2007; Midoux et al., 2008). After endocytosis, polymer nanoparticles can buffer the pH of endosome and trigger the proton sponge effect (Wojnilowicz et al., 2019). The changed osmotic pressure leads to endosome rupture and nanoparticle release. In the cytoplasm, polymer nanoparticles release polynucleotides *via* degradation. The duration and dosage of polynucleotides delivered into the cell can be adjusted by tuning the degradation rate.

Polymer vehicles have been used for polynucleotide delivery into mammalian cells. For example, siRNAs delivered by polymer nanoparticles inhibited tumor growth in *in vitro* and *in vivo* models (Pillé et al., 2006; Bartlett and Davis, 2008). For cardiac diseases, delivering siRNAs targeting leukocyte recruitment-related genes *via* PEI nanoparticles lowered inflammatory response and preserved cardiac function in a mice model of myocardial infarction (Sager et al., 2016). A major concern of polymer nanoparticles is cytotoxicity (Lv et al., 2006; Kargaard et al., 2019). For instance, PEI compromises membrane integrity and activates mitochondria-mediated apoptotic programs in human cell lines (Moghimi et al., 2005). Thus, further investigations are required to determine the safe dose of polymer vehicles.

#### 3.2.2 Dendrimers

Dendritic molecules were first synthesized in 1978 as a platform for drug delivery (Buhleier et al., 1978). A dendrimer consists of a central core, a hyperbranched mantle, and a corona (Figure 2E). The dendrimer employed for polynucleotide delivery is usually positively charged. Poly(amidoamine) (PAMAM) is a popular dendrimer for drug delivery (Figure 4). The branches in PAMAMs are terminated with cationic groups. Thus, polynucleotides can bind to PAMAM through electrostatic forces (Abedi-Gaballu et al., 2018). The mechanisms of dendrimer internalization and metabolism have not been fully understood. Studies have suggested that dendrimers are internalized through endocytosis and be degraded in lysosomes (Zhang et al., 2016; Morris et al., 2017; Chowdhury et al., 2018). Cationic dendrimer can destabilize the endosome membrane and then enter the cytoplasm. In the cytoplasm, polynucleotides are released slowly *via* dendrimer degradation.

Polynucleotide delivery using dendrimers has been applied in many gene therapy studies. Delivering microRNA-150 using PAMAM molecules ameliorated acute myeloid leukemia in mice (Jiang et al., 2016). For heart-related applications, perfusing dendrimers carrying the β-galactosidase gene into the coronary artery led to protein expression in myocytes for 7–14 days (Wang et al., 2000). In addition, intramyocardial injection of dendrimers carrying the relaxin gene promoted left ventricular systolic function in rats model of myocardial infarction (Lee et al., 2016). Notably, long-term delivery of PAMAM impaired the recovery of cardiac function after ischemia/reperfusion in rats (Babiker et al., 2020). These results indicate that dendrimers can deliver mRNA into cardiac cells; however, their safety requires further evaluation.

#### 3.2.3 Biopolymers

The primary cytotoxic concerns regarding synthetic polymer encapsulation of RNAs are significantly ameliorated by substituting in biopolymers as the basis for an RNA complex delivery system. Like the commonly used synthetic polymers, these compounds are typically arranged in a linear fashion. Silk fibroin, alginate, chitosan, gelatin, and hyaluronic acid have all been found to be effective in encapsulating polynucleotides for particle-mediated delivery. Silk-based delivery of RNA species has been designed in recent years. Of note, the source organisms (silkworm cocoon; spider) are amenable to genetic engineering to modify the peptide sequence of the silk fibroin vehicle for stability of nucleotide binding (poly-lysine motif), transfection efficiency by cell membrane destabilization, and cellular targeting (Florczak et al., 2020). With regards to alginates, studies have shown them to capable of entraining siRNA. In addition, alginate nanoparticle carriers have also demonstrated good cellular uptake and *in vitro* transfection in multiple cell types including neonatal rat cardiac fibroblasts and hepatocellular carcinoma lines (Goldshtein et al., 2019; Alallam et al., 2020). *In vivo* use has also shown promising results, with controlled release of siRNAs from PEI-alginate complexed particles reducing expression of novel tumor suppressor protein, nischarin, and improving motor function recovery in a rat spinal cord injury model (Ding et al., 2017).

Chitosan has also received interest as a nanoscale carrier due to its intrinsic ability to electrostatically complex with polynucleotides. Both siRNAs and miRNAs can be encapsulated and used to interfere with expression *in vitro* and *in vivo* (Ballarín-González et al., 2013; Nguyen et al., 2019). Despite its similarity to synthetic polymers such as PEI, which electrostatically interacts with nucleotides, these chitosan carriers show robust biocompatibility with low cytotoxicity in comparison (Berthold et al., 2010; Delas et al., 2019). Modified chitosan oligosaccharides and other chitosan systems, such as those including gelatin, have been investigated for their potential in further augmenting chitosan polyplexion’s inherent benefits (Lallana et al., 2017; Liang et al., 2020). In combination with gelatin and epigallocatechin gallate, chitosan-based particles also achieved silencing efficiencies greater than those of Lipofectamine 2000 (Zhou et al., 2022).

Likewise, nanoparticles with core polymers primarily of hyaluronic acid and gelatin have also demonstrated viability as polynucleotide carriers. In line with many of the other biopolymeric carrier systems demonstrated, CD44 targeted hyaluronic acid particles have successfully delivered siRNA for effective silencing of MDR1, a potent mutation for drug resistance in ovarian cancer cells (Yang et al., 2015). Gelatin has also shown promise as a carrier for large polynucleotide strands and, coupled with its strong biocompatibility and versatility in loading and delivery, would seem to be an ideal candidate (Morán et al., 2017). However, while it can effectively encapsulate and deliver mRNA to murine pre-osteoblast cells *in vitro*, gelatin nanoparticles have yet to demonstrate cellular translation of the delivered mRNA, presumably due to low endosomal escape (Andrée et al., 2022). Despite its seeming promise, greater control would provide the augmentation needed to further gelatin as the carrier of a large polynucleotide therapy. As introduced above, surface modifications can address nucleotide absorption to particle delivery. Similarly, modifications to gelatin nanoparticles with cationic polymer PEI has been shown to increase endosomal escape by endosome osmotic swelling (Huang et al., 2011b). PEI-modified gelatin nanoparticles showed efficacy for protein and plasmid delivery in pre-osteoblastic cells in the dish and *in vivo*.

### 3.3 Viral vehicles

Viral vehicles are not designed for mRNA delivery. Nevertheless, they can transfer mRNA templates, usually in the form of DNA fragments, into mammalian cells. Thus, viral vehicles are briefly introduced in this section. Detailed reviews can be found in articles focusing on viral vectors for gene therapy (Bulcha et al., 2021; Raguram et al., 2022).

#### 3.3.1 Adenoviral vector

Adenoviral vector is derived from adenovirus. It consists of a protein shell, a protein core, and a 30 kb linear DNA genome (Figure 2F). In the body, adenovirus adheres to the cell membrane and then enters the cell *via* vesicle-mediated engulfment (Lonberg-Holm and Philipson, 1969). After entering a cell, the protein core migrates to the nucleus and releases the genome within 2 h. Studies have indicated that adenoviral vectors can transfect cardiac cells in neonatal rats (Stratford-Perricaudet et al., 1992). Intravenous injection of adenoviral vectors carrying the lacZ gene resulted in the expression of β-galactosidase in many organs including the heart. Approximately 0.2% of neonatal cardiac cells were transfected on day 15 after injection. The transfected cardiomyocytes expressed β-galactosidase for at least 12 months. The results suggest that genes delivered by adenoviral vectors are expressed for a long term in cardiac cells. In preclinical trials, delivering adenoviral vectors carrying VEGF gene improved angiogenesis and fractional wall thickening in swine hearts after-ischemic cardiac injury (Mack et al., 1998). A follow-up clinical trial demonstrated that this therapy is safe for human beings (Rosengart et al., 1999). The effectiveness of this therapy for patients is under investigation.

#### 3.3.2 Adeno-associated viral vector

**FIGURE 5:**
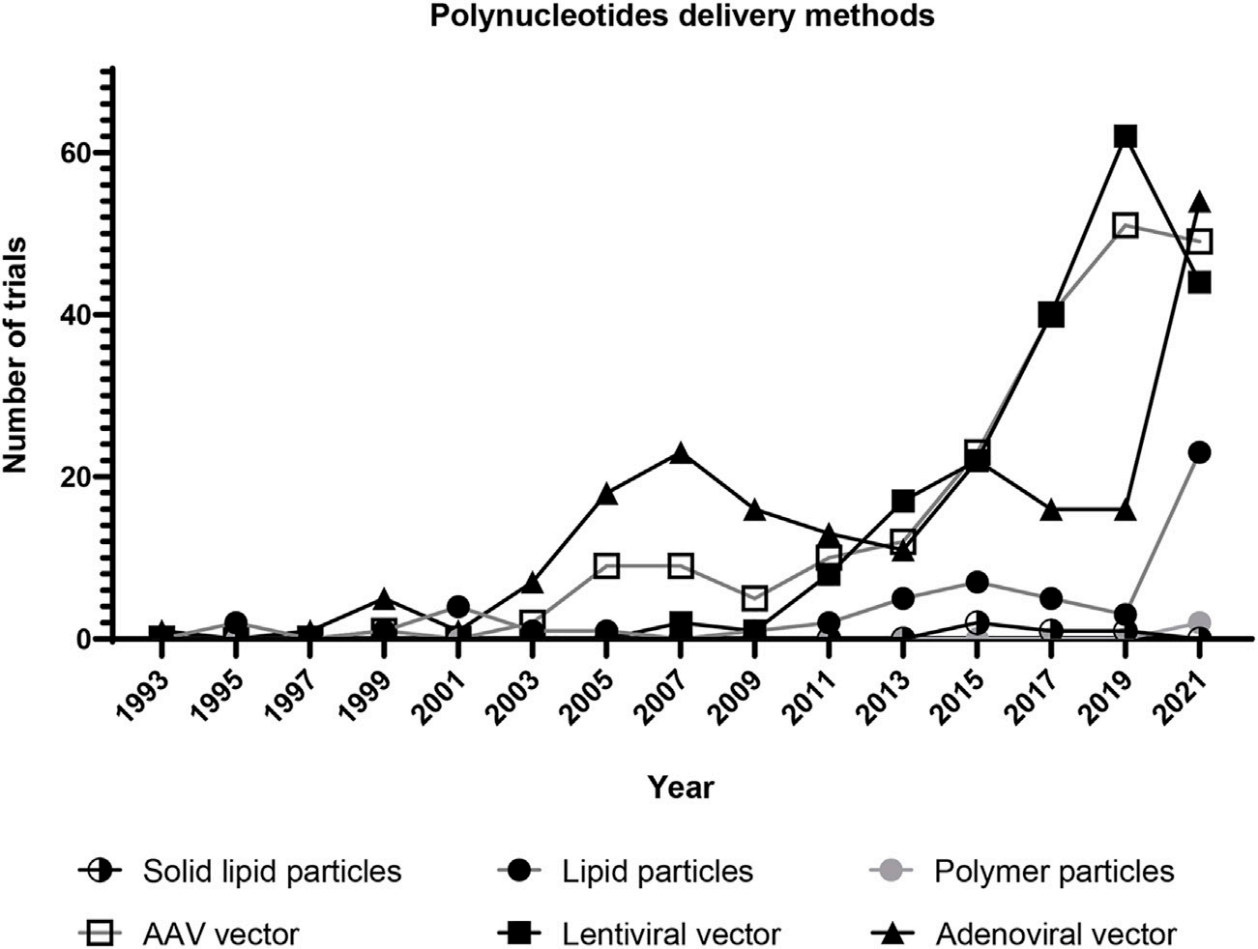
Clinical trials of gene therapy using selected vehicles for polynucleotide delivery. Each dot represents the number of clinical trials in 2 years. The trials using lentiviral, adenoviral, and AAV vectors have grown since 1995. The trials using lipid nanoparticles have significantly increased since 2019. This is probably due to the development of COVID-19 vaccines. (Solid lipid particle data were collected by searching for “solid lipid nanoparticle” and “nanostructured lipid carrier” in the *Other terms* search field on ClinicalTrials.gov. Lipid particle data were collected by searching for “liposome gene” and “lipid nanoparticle” in the *Other terms* search field. Polymer particle data were collected by searching for “polymer nanoparticle” and “dendrimer” in the *Other terms* search field. Lentiviral vector data were collected by searching for “lentiviral vector” in the *Other terms* search field. Adenoviral vector data were collected by searching for “adenoviral vector” in the *Other terms* search field. AAV vector data were collected by searching for “AAV vector” in the *Other terms* search field. All data was collected from ClinicalTrials.gov on 22 April 2022).

AAV consists of a 26 nm protein shell and a 4.7 kb single-stranded DNA (Figure 2G). Because AAV needs adenovirus to replicate, no pathogenic effects have been observed in AAV-transfected cells (Wang et al., 2019). To transfect a cell, AAV first adheres to cytoplasm receptors such as the AAV receptor, and then enters the cell *via* endocytosis (Pillay et al., 2016). In the cytoplasm, AAV is transported to the perinuclear region in endosomal compartments, and then escapes from the endosome through acidification events (Nonnenmacher and Weber, 2012; Xiao and Samulski, 2012). The protein core eventually enters the nucleus through the nuclear pore complex (Nicolson and Samulski, 2014). The transfection efficiency of AAV vectors is almost ten times higher than non-viral techniques (Djurovic et al., 2004). AAV-mediated gene therapy has been investigated in clinical trials for genetic disorders associated with the brain, spinal cord, eyes, livers, and muscle diseases (Wang et al., 2019). For cardiac disease, percutaneous administration of AAV1 vectors carrying sarcoplasmic reticulum calcium ATPase gene into heart failure patients decreased 3-year mortality compared to the placebo group (Jessup et al., 2011; Zsebo et al., 2014). In addition to clinical trials, promising results have been observed in animal models. Intramyocardial injection of chimeric AAV vectors carrying genes for fibroblast reprogramming increased heart regeneration in mice myocardial infarction model (Yoo et al., 2018). Because of their low toxicity and high gene delivery efficiency, AAV vectors have become one of the most popular vehicles for gene therapy in clinical studies (Figure 5).

#### 3.3.3 Lentiviral vector

Lentivirus is a subclass of retroviruses. The lentiviral vector is derived from the human immunodeficiency virus (HIV). It contains a fractional set of the HIV genome and helper genes (Figure 2H) (Dull et al., 1998). After delivering into the body, lentivirus enters the cytoplasm *via* membrane fusion or endocytosis. The viral RNA is converted to double-stranded DNA through reverse transcription in the cytoplasm; and the DNA is then transported to the nucleus by microtubules (de Rijck et al., 2007; Escors and Breckpot, 2010). After being inserted into the host genome, the lentiviral genome can be expressed in mammalian cells for months (Naldini et al., 1996). Gene therapy *via* lentiviral vectors has been applied *in vivo* for cardiac diseases. Delivering lentiviral vectors carrying the angiotensin gene into myocardium reduced left ventricular remodeling in rats myocardial infarction model (Qi et al., 2011). Delivering the sarcoplasmic reticulum Ca^2+^ ATPase gene also promoted cardiac repair in rats (Niwano et al., 2008). In addition, editing genes using lentiviral vectors successfully induced mesenchymal-like cells to cardiomyocyte differentiation (Koyanagi et al., 2010). The transfected cells can be used to stimulate cardiac repair. Due to their high transfection efficiency, lentiviral vectors have become a popular tool for gene editing in clinical trials (Figure 5). However, further optimization is required to solve safety issues such as insertional mutagenesis and off-target effects.

### 3.4 Route of administration

The route of administration is a crucial factor in mRNA therapy for cardiac diseases. Parenteral injection is the most common method for polynucleotide delivery into the heart (Table 2). Among all the 34 clinical trials for cardiac gene therapy, 11 used intracoronary injection, 13 used intramyocardial injection, 4 used intravenous infusion, and 5 used subcutaneous injection. Notably, 1 trial initiated in September 2022 utilized polymer hydrogel to deliver the vehicle (Table 2) (Liu et al., 2017). It suggests that localized and sustained delivery of polynucleotides *via* biomaterials has become an option for cardiac diseases in patients.

**FIGURE 6:**
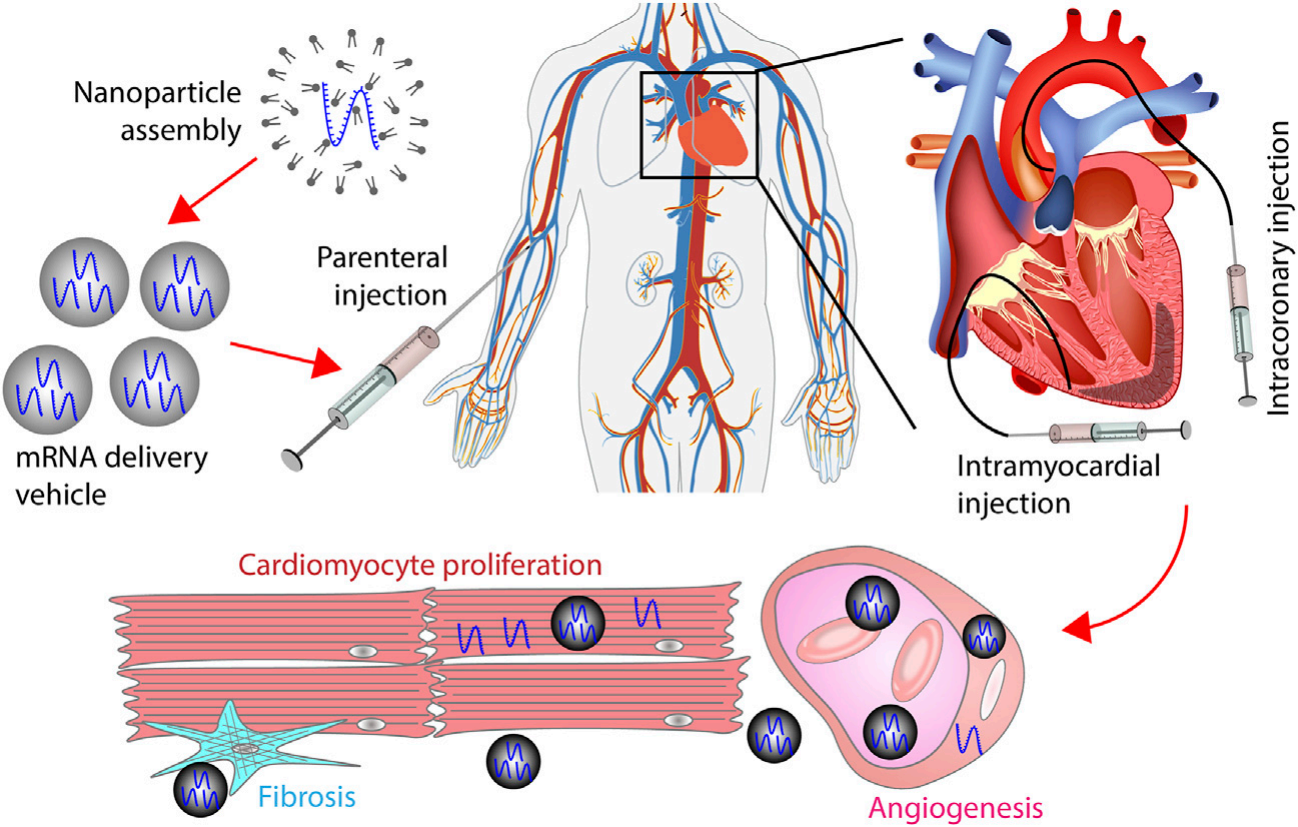
mRNA therapy for myocardial infarction. mRNAs encapsulated by vehicles are administrated into the heart through intravenous, subcutaneous, intracoronary, or intramyocardial injection. The route of administration can affect the percentage of mRNAs that reach the heart. In the heart, vehicles extravasate from the vessel to the extracellular space. Afterward, vehicles are internalized by cardiomyocytes, fibroblasts, endothelial cells, or smooth muscle cells. mRNAs delivered by vehicles promote cardiac regeneration by stimulating cardiomyocyte proliferation, increasing angiogenesis, and inhibiting fibrosis.

Subcutaneous injection and intravenous infusion are feasible for drugs that target biomolecules in the circulation system (Figure 6). For example, the antisense oligonucleotide (ASO) that lowers blood lipoprotein level was delivered *via* subcutaneous injection in patients. Intramyocardial and intracoronary injections are preferred for heart-specific delivery (Figure 6). In intracoronary injection, polynucleotides are administrated through a catheter placed in the coronary artery. In intramyocardial injection, a catheter is inserted into myocardium. A challenge for intramyocardial and intracoronary injections is that cardiac catheterization can cause severe adverse effects such as death and myocardial infarction. Although the incidence of major complications is low, routine catheterization for drug delivery can significantly increase the risk of major complications (Manda and Baradhi, 2022). Delivering mRNA and its vehicle using hydrogel is a relatively new method for localized drug delivery. Hydrogels can be implanted into the heart through surgery or interventional procedures. An advantage of this method is that mRNA can be consistently delivered into the heart for a long time.

Improving the therapeutic efficacy of systemically delivered mRNA drugs could be achieved by employing vehicles with high heart-specificity. The available viral and non-viral vehicles exhibit low specificity to cardiac cells. In viral vehicles, the recombinant AAV serotype 9 (rAAV9) appears to be the most cardiotropic (Williams et al., 2010; Pacak and Byrne, 2011). Notably, over 20-times more vector copies were found in the liver compared to the heart following systemic delivery of rAAV9 (Inagaki et al., 2006). The factors controlling the heart-specificity in viral vehicles remain unknown. However, replacing the capsid components and employing cardiac-specific promoters may increase the heart-specificity of AAV vehicles (Williams et al., 2010; Bilal et al., 2021). For non-viral vehicles, surface modification is capable of improving heart-specificity. Nevertheless, the surface-modified viral and non-viral vehicles are still subject to nonspecific sequestration by the reticuloendothelial scavenger cells (Sørensen et al., 2012). As a result, a significant portion of systemically delivered vehicles is sequestrated before reaching the target. Preclinical studies have demonstrated that transiently blocking the scavenger receptors or masking the liver sinusoidal wall lowers the nonspecific sequestration (Haisma et al., 2008; Dirisala et al., 2020). Ongoing investigations may reveal more methods for reducing nonspecific sequestration.

## 4 Clinical application of gene therapy for cardiac diseases

mRNA therapy for cardiac diseases has not been investigated in clinical trials. The superclass of mRNA therapy, gene therapy, is discussed here. The signaling pathways utilized in gene therapy are also potential targets of mRNA therapy. The number of gene therapies under development for cardiac diseases has grown significantly in the past 20 years (Table 2). Among the clinical trials recorded on CliniclTrials.gov, 22 out of 34 trials were started between 2012 and 2022. Most of the clinical trials are ongoing. However, some of the completed or terminated trials have released results. MYDICAR, a gene therapy drug that has been investigated in clinical trials since 2007, lowered the rate of non-terminal events in chronic heart failure patients (Zsebo et al., 2014). Gene therapies targeting Adenylyl cyclase 6 (ADCY6) promoted left ventricular ejection fraction at week 4 in symptomatic heart failure patients (Hammond et al., 2016). VM202, a naked DNA plasmid, increased stress perfusion in ischemic heart disease patients when applied as an adjunct therapy to coronary artery bypass grafting (Kim et al., 2013). Transferring the human FGF4 gene into angina pectoris patients improved myocardial perfusion and exercise treadmill testing score (Grines et al., 2002; Grines et al., 2003; Henry et al., 2007). Mipomersen, an ASO drug approved by FDA, significantly lowered the levels of atherosclerotic lipoprotein in hypercholesterolemia patients (Raal et al., 2010; Thomas et al., 2013). Together, the clinical data has demonstrated that gene therapies *via* engineered viral vectors or naked polynucleotides are effective for patients. However, so far none of the clinical studies have led to therapeutics for myocardial infarction.

## 5 Challenges and future directions

Toxicity is a major concern revealed by clinical trials of gene therapy for cardiac diseases. The completed phase I and phase II trials have demonstrated that delivering DNA plasmids or viral vehicles does not have acute toxicity for human beings. However, the chronic effects should not be ignored. For instance, long-term administration of mipomersen can induce liver steatosis and less serious adverse effects, including nausea, headache, and flu-like symptoms (Hashemi et al., 2014). For safety issues, mipomersen has to be prescribed under a risk management plan. Managing the risk of mRNA therapy is relatively easy because RNAs are metabolized within a few weeks. Thus, the side effects can be attenuated by stopping drug administration. Managing the risks of long-term gene therapy is challenging because the incorporated genes are expressed for months to years. As a result, the adverse effects may last for a long period or, in the worst scenario, lead to death. For instance, uncontrolled cardiomyocyte proliferation induced by AAV-mediated gene therapy led to sudden death in swine myocardial infarction model at week 7 post-treatment (Gabisonia et al., 2019). Thus, the dosage and delivery method need to be reviewed before large-scale clinical studies.

Another hurdle revealed by clinical trials is that the therapeutic efficacy of gene therapy drugs is not satisfying. For example, MYDICAR has been investigated in 6 clinical trials in the United States but failed to improve the clinical course of patients with heart failure (with reduced ejection fraction) in phase II trials (Jessup et al., 2011). BioBypass, an adenoviral vector carrying the VEGF-121 gene, was safe for refractory myocardial ischemia patients but failed to improve exercise capacity and myocardial perfusion in the phase II trial (Kastrup et al., 2011). In another phase II trial involving VEGF, delivering plasmids carrying human VEGF-A165 and bFGF into the myocardium of patients with refractory heart ischemia did not attenuate symptoms (Kukuła et al., 2011; Kukuła et al., 2019). In addition to VEGF treatment, no significant improvement has been observed in clinical trials investigating gene therapies targeting HGF, SDF-1, and FGF4. Improving the therapeutic efficacy of gene therapy in clinical trials remains an unmet need for cardiac diseases.

Exploring new targets and developing new delivery vehicles for cardiac cells will benefit gene therapy for cardiac diseases. The potential targets are introduced in prior sections. Some of the targets, for example, VEGF and FGF, have been investigated in clinical trials. Future studies investigating signaling pathways that regulate cardiomyocyte proliferation, fibroblast differentiation, and endothelial cell migration may reveal effective targets for cardiac gene therapy. In addition, developing vehicles for high-efficient polynucleotide delivery into target cells will improve the therapeutic efficacy of gene therapy. Further evaluation of molecular composition of lipid and polymer based vehicles have the potential to improve therapeutic efficacy by lowering the immune response. The cell specificity and delivery efficiency of available polynucleotide delivery methods are low. Increasing the cell specificity by surface modification may result in targeted polynucleotide delivery. The delivery efficiency is determined by several factors including the internalization rate and endosome escape rate. These factors can be modulated by changing the base materials. Besides, chemical modification of the nanoparticle surface may facilitate endocytosis or destabilize endosome membranes.

In conclusion, mRNA therapy is a relatively new strategy for cardiac diseases. Clinical and pre-clinical studies have shown encouraging results. Meanwhile, the studies also revealed issues including low therapeutic efficacy and heart-specificity. mRNA therapy for cardiac diseases can be optimized by screening for new targets and developing strategies for heart-specific mRNA delivery. Interdisciplinary investigations of biomaterials and mRNA designs will lead to commercialized medicines for myocardial infarction.
